# Effect of fiber addition on strength and toughness of rubberized concretes

**DOI:** 10.1038/s41598-024-54763-w

**Published:** 2024-02-22

**Authors:** Sameh Mohamed, Hesham Elemam, Mohamed H. Seleem, Hossam El-Din M. Sallam

**Affiliations:** 1 Materials Engineering Department, Faculty of Engineering, Zagazig University, Zagazig, 44519 Egypt; 2 Civil and Environmental Engineering Departement, University of Missouri, Columbia, MO 65202 USA

**Keywords:** Rubberized concrete, Fiber reinforced concrete, Mechanical properties, Impact energy, Civil engineering, Mechanical properties

## Abstract

In this paper, an experimental study was conducted to examine the static and dynamic behaviors of rubberized fiber-reinforced concrete (RFRC). Crumb rubber was partially replaced from sand at volume fractions of 0%, 5%, 10%, 15%, and 20%. Steel fibers (SFs) with fiber volume fractions (Vf%) of 0%, 0.5%, 1%, and 1.5% were used for the production of FRCs, while polypropylene fiber (PPF) with Vf% = 0.4% was adopted to produce others FRCs. A combination of 0.4% PPF and 1% SF was used for hybrid FRC. The static properties were evaluated through compression, indirect tension, and flexural tests. However, the drop weight impact test was conducted to assess the dynamic property by estimating the impact energy. It was observed that the replacement of sand with rubber reduced all mechanical properties of concrete. In the case of RFRC, a reduction in compressive strength, compared to samples without fibers, was noted, and this reduction increased with higher Vf%. Both toughness indices and fracture energy were affected slightly by increasing rubber percentages while markedly increased with higher Vf%. However, adding rubber and/or fibers enhanced the impact energy of concrete.

## Introduction

Due to its affordability, abundant raw material supply, and excellent compressive strength, concrete is one of the most extensively employed construction materials worldwide. The fundamental elements of concrete, including sand and gravel combined with a hydraulic binder and water, have been used in construction practices since ancient Egypt^1–3^. The original concrete formula has been modified to incorporate various additives or admixtures (such as fibers, superplasticizers, and industrial byproducts) to enhance concrete strength, ductility, durability, workability, and sustainability. Advancements in concrete technology have made it possible to select appropriate materials and design a concrete mix that fulfills performance requirements while keeping economic and environmental costs as low as possible. Traditional concrete, characterized by its high rigidity and stiffness (resulting in low toughness), can improve toughness, ductility, energy dissipation capacity, and impact resistance by adding rubber (RU). This is achieved through the partial substitution of aggregates, resulting in what is known as rubberized concrete (RUC)^2,3^. The unique properties of RUC render it a promising material for various architectural applications, encompassing highway pavements, impact-resistant wall panels, crash barriers, and slabs on grade^4^. Additionally, using RU derived from end-of-life tires enhances the eco-friendliness and cost-efficiency of concrete production. Environmental challenges stemming from the accumulation of millions of discarded tires worldwide^5^ are effectively addressed through the incorporation of RU from waste tires, as these tires do not readily decompose when subjected to landfill treatment^6^.

Recycling worn tire RU is essential for making concrete more environmentally friendly and cost-effective, as it prevents the accumulation of millions of discarded tires worldwide^4,5^. It is anticipated that by 2030, approximately 1.2 billion worn tires will be in use on the roads^7^. The persistence of tire RU in landfills, where it does not readily decompose, poses a significant environmental challenge. In the early 1990s, waste tire RU particles were included in cement concrete to enhance its toughness, impact resistance, and properties related to sound insulation, energy absorption, and energy consumption^8,9^. Scholars have undertaken recent research efforts to thoroughly investigate the influence of RU content, surface conditions, and mechanical attributes of concrete^10^. It has been observed in previous studies that as the proportion of RU increases, RUC experiences a reduction in both strength and modulus of elasticity^11^. Additionally, substituting natural aggregates with RU particles substantially decreases concrete's mechanical properties^12,13^. For instance, a 100% RU content can lead to a reduction in compressive strength of up to 90%. When the replacement ratio of RU particles reaches 50%, RUC's compressive strength and elastic modulus may decrease by as much as 70%^14,15^. To mitigate significant reductions in strength, it is recommended that the replacement ratio of RU particles should not exceed 25%^16^ and should ideally remain within the range of 25% to 30% of the total aggregate volume^17^.

The exploration of RUC composites in pavement applications was conducted by Phuong et al.^18^. Emphasis was placed on the superiority of RUC composites over conventional concrete composites, attributing this superiority to their higher strain capacity, enhanced freeze–thaw resistance, reduced propensity for crack initiation to mitigate shrinkage, and improved sound insulation. Khatib and Bayomy^11^ investigated the effect of replacing coarse aggregate with RU block, resulting in improved toughness and energy absorption, albeit at the expense of reduced compressive and splitting tensile strength. According to Topçu^19^, including large RU particles significantly enhances concrete's impact resistance, particularly in hammer drop tests. Park et al.^20^ partially replaced the sand with RU by 0% to 20% of the volume in RUC. Their findings indicated a gradual decrease in compressive strength as the RU content increased.

Recent research suggests that steel fibers (SF) can be incorporated into RUC to enhance its properties^21–24^. Pham et al.^24^ found that the combination of ultra-high-performance concrete (UHPC) and rubber powder could provide acceptable compressive strength than other rubberized concrete. Experiments have demonstrated that combining steel fibers with RU particles can improve concrete's flexural and ductility characteristics^25^. Compressive tests and splitting tensile tests conducted by Eisa et al.^26^ revealed that adding 1.0% steel fiber to RUC with 10% RU particle volume substitution resulted in an 11% increase in compressive strength and a 41% increase in tensile strength. An experimental study by Noaman et al.^21^ investigated the compression toughness of rubberized fiber-reinforced concrete (RFRC) with varying RU content. Abaza and Hussein^22^ that SF reinforcement transforms RUC from a brittle to a ductile failure mode. Incorporating crumb RU and SF into RFRC significantly enhances flexural toughness due to the bridging effect of the fibers. However, the interaction between RU and SF regarding flexural strength and fracture characteristics remains unclear. Liu^27^ conducted a study varying the RU content in RUC, finding that adding SF increased the concrete's strength.

Moreover, SF incorporation significantly improves RUC's toughness index and crack resistance. Ismail and Hassan^25^ conducted splitting tensile and flexural tests on steel fiber-reinforced RUC, demonstrating that the flexural and splitting tensile strength of steel fiber-reinforced concrete (SFRC) exceeded that of conventional RUC. The presence of steel fiber also enhanced the toughness of SFRC. Ngo et al.^28^ proposed a new hybrid concrete joint for corrosion damage mitigation. They found that applying the modified concrete model well captured the failure mode up to the peak load. Ha et al.^29^ investigated the effect of using alternative cementitious constituents on the compressive performance of ultra-high-performance concrete (UHPC) for both static and dynamic conditions. They concluded that UHPC was not strain rate sensitive using alternative cementitious constituents. Sukontasukkul et al.^30^ experimentally investigated the ability of a crumb RUC layer to enhance the impact resistance of an SFRC layer. The test results showed that the crumb RUC layer effectively absorbed impact energy, preventing damage to the SFRC layer. Because of the limitations in prior research and the unaddressed aspects in this field, which looked into steel fibers and RUC separately, the main objective of this paper is to explore the combined effects of SF and RUC on the static and dynamic behaviors of concrete.

Several research endeavors have separately explored the performance of steel fiber concrete and rubberized concrete. However, this paper takes a comprehensive approach by investigating the combined influence of steel fiber and rubber on the static and impact resistance of concrete. The study not only delves into the mutual effects but also scrutinizes how introducing steel fibers with different volume fractions with the optimal percentage of rubber improves the impact resistance of concrete. This dual-focus analysis provides a more nuanced understanding of the intricate interactions between steel fiber and rubber in enhancing concrete properties in terms of static strength and resistance to impact forces.

## Experimental program

### Experimental program

The present experimental program consisted of twenty-four mixes of RFRC, as listed in Table 1. The RUC mixes were prepared in the initial four sets, with fine RU particles incorporated at volume fractions of 0%, 5%, 15%, and 25% as a partial replacement for fine aggregate to be used as control mixes for comparison. Subsequently, SF and polypropylene fiber (PPF) were added separately and combined (hybrid) to the same previous four sets to produce other sets of RFRCs. In the case of SF, three different fiber volume fractions (Vf%), i.e., 0.5%, 1%, and 1.5% were adopted, while 1% SF and 0.4% PPF were combined to make a hybrid RFRC. The PP RFRC was made by adding 0.4% PPF, as listed in Table 1. This systematic approach enabled the comprehensive examination of the influence of different fiber types, volumes, and RU content on the properties of concrete mixtures.

**Table 1:** Mix code and experimental program conducted in the present work.

Mix no.	Mix code	RU		SF		PPF	
		%	kg/m^3^	%	kg/m^3^	%	kg/m^3^
1	RU0*	0	–	0	–	0	–
2	RU5	5	12	0	–	0	–
3	RU15	15	36	0	–	0	–
4	RU25	25	60	0	–	0	–
5	SF0.5RU0**	0	–	0.5	39.5	0	–
6	SF0.5RU5	5	12	0.5	39.5	0	–
7	SF0.5RU15	15	36	0.5	39.5	0	–
8	SF0.5RU25	25	60	0.5	39.5	0	–
9	SF1RU0	0	–	1	79	0	–
10	SF1RU5	5	12	1	79	0	–
11	SF1RU15	15	36	1	79	0	–
12	SF1RU25	25	60	1	79	0	–
13	SF1.5RU0	0	–	1.5	118.5	0	–
14	SF1.5RU5	5	12	1.5	118.5	0	–
15	SF1.5RU15	15	36	1.5	118.5	0	–
16	SF1.5RU25	25	60	1.5	118.5	0	–
17	PP0.4RU0***	0	–	0	–	0.4	3.6
18	PP0.4RU5	5	12	0	–	0.4	3.6
19	PP0.4RU15	15	36	0	–	0.4	3.6
20	PP0.4RU25	25	60	0	–	0.4	3.6
21	SF1PP0.4RU0	0	–	1	79	0.4	3.6
22	SF1PP0.4RU5	5	12	1	79	0.4	3.6
23	SF1PP0.4RU15	15	36	1	79	0.4	3.6
24	SF1PP0.4RU25	25	60	1	79	0.4	3.6

*rux, where x is the percentage of fine rubber. **SFy, where y is the steel fibers volume fraction. ***PPz, where z is the polypropylene fibers volume fraction.

### Materials

**Figure 1:**
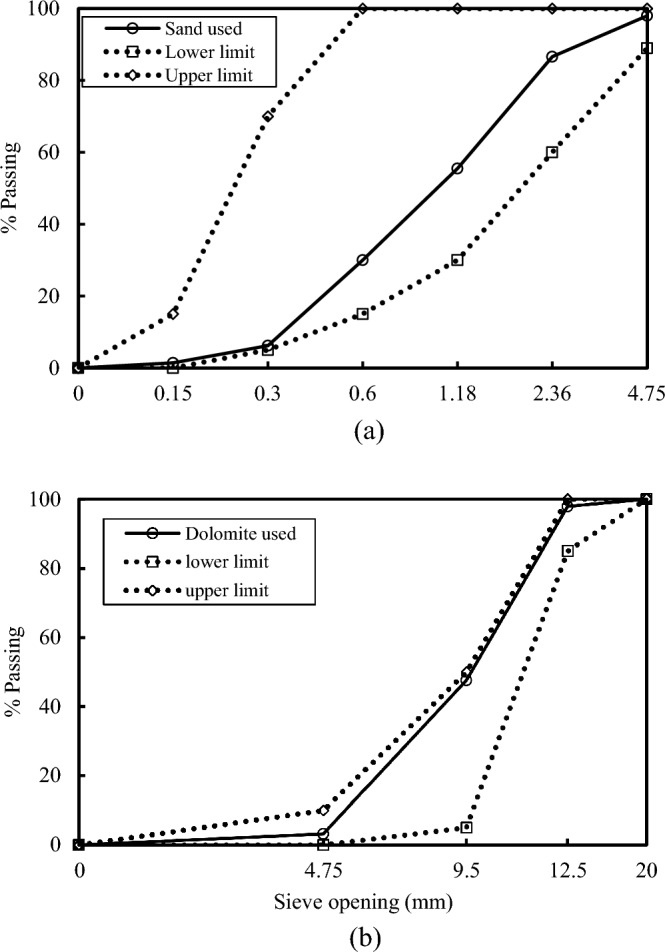
Grading curves for (**a**) Sand and (**b**) Dolomite.

Grade 42.5N Ordinary Portland Cement was employed in the present study. Dolomite, characterized by a maximum aggregate size of 12.5 mm, a specific gravity of 2.54, and a compacted density of 1.45 tons/m^3^, was utilized as the coarse aggregate. The fine aggregate consisted of siliceous sand with a specific gravity of 2.59, a compacted density of 1.72 tons/m^3^, and a fineness modulus of 2.85. Grading curves conforming to the limits outlined in BS 822:1992 for both the coarse and fine aggregates used in the study are depicted in Fig. 1.

The tire recycling process for crumb rubber used in concrete involves shredding, separating textile and steel components, and generating rubber grains. Subsequently, the crumb rubber undergoes classification based on various sizes to ensure its suitability for specific applications. The production of crumb rubber in different grain sizes includes grinding processes conducted at ambient temperature with and without wet conditions, high temperature (around 130 °C), and freezing temperature. The grinding methods aim to produce rubber particles suitable for diverse applications, employing different temperature conditions and milling techniques. Ground tire RU particles, free from tire strings, were used. The RU possessed a reasonably uniform size, passing through a 4.75 mm sieve and being retained on a 1.18 mm sieve. The rubber is cleaned and rinsed with water, then set aside until its surface is completely dry. The approximate compacted density of the used RU was 0.497 tons/m^3^. For the production of the FRC with SF, hooked-end SF measuring 35 mm in length and 0.80 mm in diameter were employed. The SF had a 7.85 tons/m^3^ density and a tensile strength of 1100 MPa. To create FRC with PPF, PPF with a length of 18 mm and a tensile strength of 600 MPa were utilized. A high-performance superplasticizer known as PANTARHIT ® PC 180 (FM), following EN 934-2^31^, was used in a ratio of 0.5% relative to the weight of cement. These materials were proportioned to generate 24 mixtures following the experimental program presented in Table 1. The cement content and the water-to-cement ratio for all combinations were maintained at 400 kg/m^3^ and 0.54, respectively.

### Test specimens

**Figure 2:**
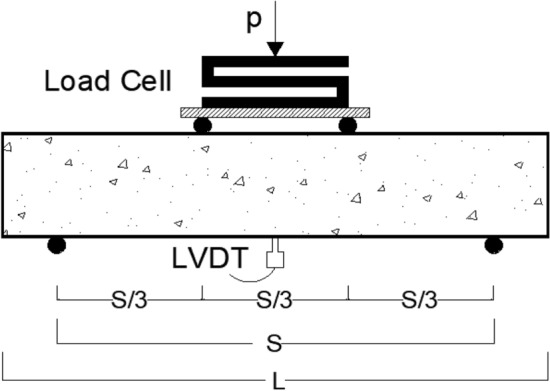
Beam specimen configuration.

**Figure 3:**
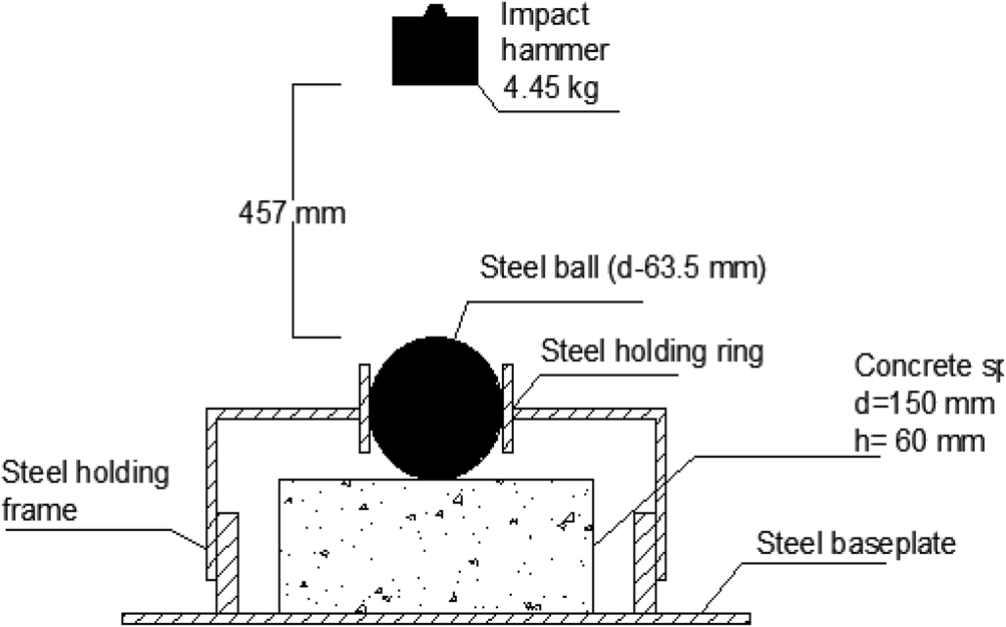
Impact test specimen configuration.

Cube specimens with dimensions of 100 × 100 × 100 mm were prepared per BS EN 12390-3^32^ for the compression test. The compressive strength of the mixture was determined by considering the average tested value of five samples. For the indirect tensile test, cylindrical specimens measuring 100 mm in diameter and 200 mm in height were prepared, following the guidelines of BS EN 12390-6^33^. The tensile strength of the mixture was estimated by averaging the test values obtained from five cylindrical samples. A beam specimen featuring cross-section dimensions (h × b) of 100 mm × 100 mm and a total span (L) of 500 mm was employed for the bending test, as per the specifications of BS EN 12390-5:2009^34^, see Fig. 2. The beams underwent four-point bending on a loaded span (S) of 400 mm, and the flexural strength of the mixture was determined by considering the average tested value of three beams. For the drop weight impact test, disc specimens with a diameter of 150 mm and a height of 60 mm were utilized following ACI committee 544.2R-89^35^, see Fig. 3. An average value was obtained from the results of five cast discs. The compression, indirect tension, and flexure tests were conducted on all 24 mixes. In the drop weight impact test, the effect of SF addition on rubberized concrete with 15% RU was examined, i.e., mixes 3, 7, 11, and 15 listed in Table 1 were implemented.

### Mixing, casting, and curing

**Figure 4:**
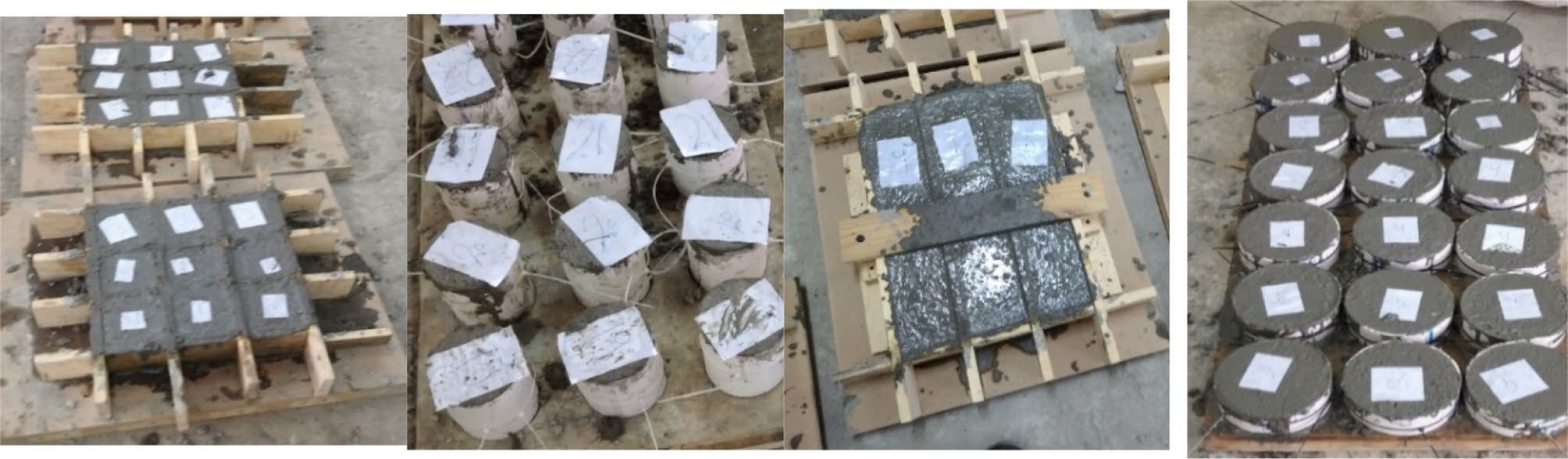
Compression, indirect tension, flexural, and impact specimens after casting.

The mixing procedures for each of the 24 mixes were executed as follows: Initially, fine aggregates, coarse aggregates, and RU were blended in the mixer for 1 min. Then, cement and 2/3 of the water were introduced and mixed for 1 min. After a total mixing time of 2 min, 1/3 of the water volume and superplasticizer were added, and mixing continued for 1 min. For mixes incorporating SF or PPF, the fibers were sprinkled into the mix after 3 min and then mixed for an additional 1 min. Cubes, cylinders, beams, and discs were cast in oil-coated molds. Subsequently, the models were filled to the top, and the tamping and tapping process was repeated. A smooth finish was achieved using a tamping rod to slide horizontally back and forth across the top. Figure 4 illustrates a set of concrete specimens for compression, indirect tension, flexural, and impact tests immediately after casting. All test specimens were removed from their molds 24 h after casting and then saturated in water tanks for 28 days.

### Instrumentation and test setup

**Figure 5:**
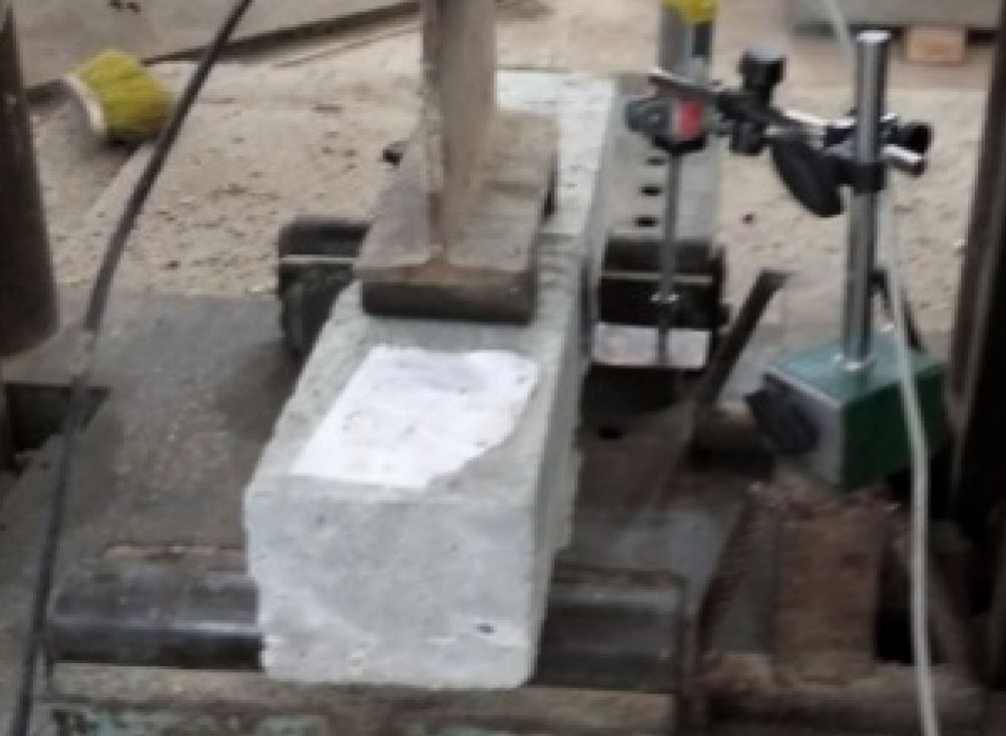
Flexure test setup.

**Figure 6:**
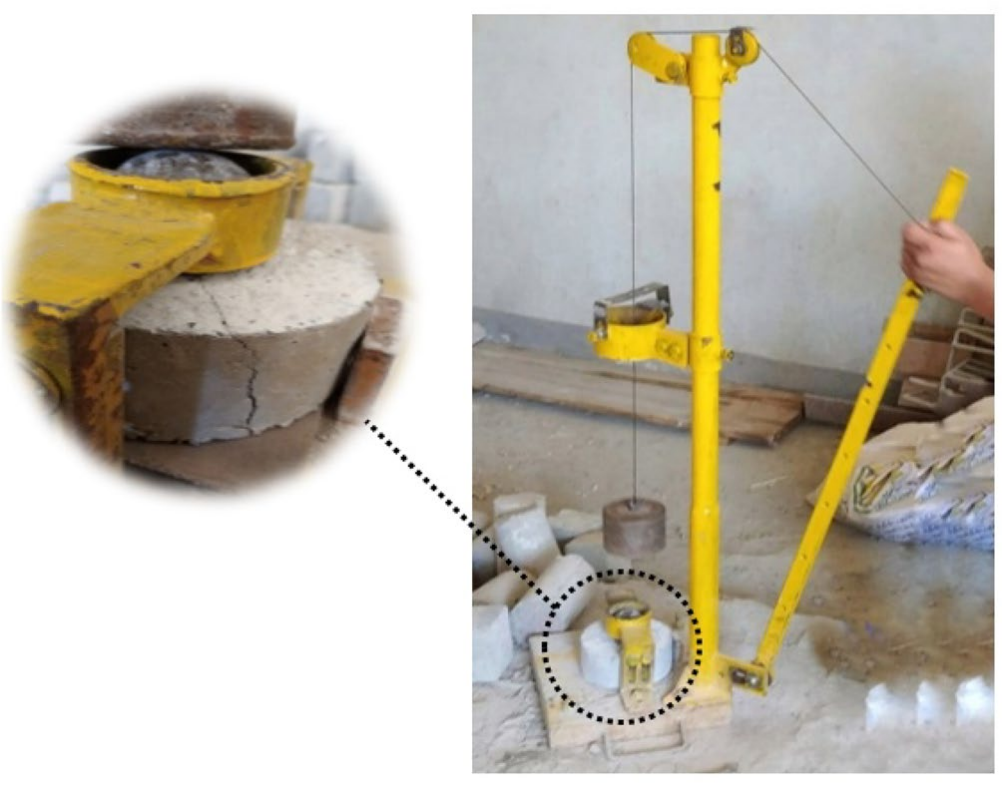
Impact test setup.

Compression and indirect tension tests were performed using a 3000 kN capacity hydraulic testing machine under load control conditions. The flexure test was performed on a hydraulic testing machine with a capacity of 1000 kN. The measurement of load was facilitated by employing a 100 kN load cell. The mid-span deflection of the beam was measured using an LVDT with an accuracy of 0.001 mm, as depicted in Fig. 5. For the impact test, a drop weight machine, as illustrated in Fig. 5, was employed. In this test, repeated blows were administered to the disc surface from a height of 457 mm, utilizing a 4.45 kg hammer that descended upon a steel ball with a diameter of 63.5 mm positioned at the center, as shown in Fig. 6.

## Results and discussion

### Compressive, splitting tensile, and flexural strengths

**Figure 7:**
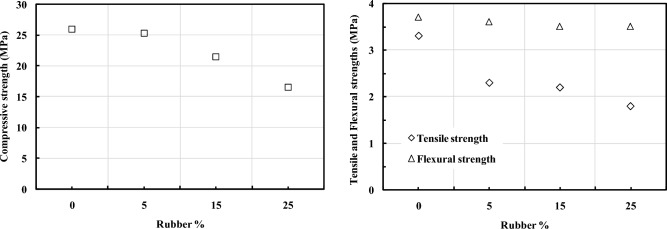
Effect of RU% on the compressive, tensile, and flexural strengths of RUC.

All results of compressive, tensile, and flexural strengths of both RUC and RFRC are listed in Table A1. In general, replacing fine aggregate with rubber decreases all the mechanical properties of concrete, while adding fibers to rubberized concrete enhances all its mechanical properties. The effect of sand replacement with varying percentages of RU (0%, 5%, 15%, and 25%) on the compressive, tensile, and flexural strengths of RUC is shown in Fig. 7. Substituting sand with RU led to a reduction in all mechanical properties. Notably, the decrease in compressive strength was minimal at 5% RU content. However, at higher RU percentages (15% and 25%), a substantial reduction became evident, registering at 17.3% and 36.5%, respectively, as illustrated in Fig. 7. This decrease in the compressive strength can be attributed to the increased porosity and the formation of weak points within RUC. These weak points arise from inadequate bonding between the RU and the concrete mixture, resulting in high internal stresses perpendicular to the direction of the applied load, as previously discussed^36,37^. The reduction in tensile strength was more pronounced for all RU percentages, reaching 29.5%, 32.6%, and 44.7% for RU percentages of 5%, 15%, and 25%, respectively. Meanwhile, the results for flexural strength indicated a modest decline, with an average decrease of 4.3% for all RU percentages.

**Figure 8:**
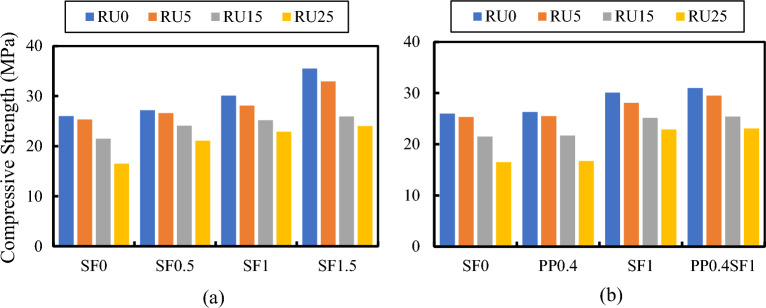
Effect of (**a**) SF% and (**b**) Type of fiber on the compressive strength of RFRC.

**Figure 9:**
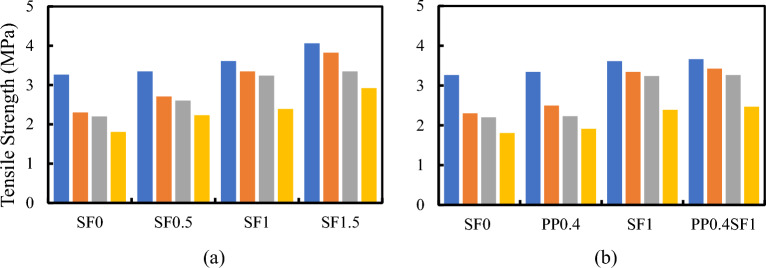
Effect of (**a**) SF % and (**b**) Type of fiber on the tensile strength of RFRC.

**Figure 10:**
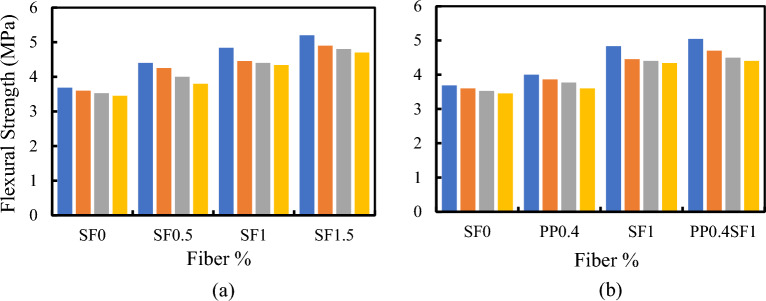
Effect of (**a**) SF% and (**b**) type of fiber on the flexural strength of RFRC.

The influence of adding fibers (SF, PPF, or hybrid fibers) on compressive, tensile, and flexural strengths is shown in Figs. 8, 9 and 10, respectively. Regardless of the RU content, increased fiber volume fraction improved all strengths. The maximum strength enhancement was observed with SF. Adding PPF enhances the mechanical properties of RUC compared to mixes without fibers. Higher strengths were attained by the hybrid fibers mixture when contrasted with the SFRC blend containing 1% SF. The increase in compressive strength attributed to including fibers in the binder matrix is ascribed to the increased stiffness of composite materials. The addition of fibers exerts a confining effect on the binder matrix, thereby mitigating the deformation of the composite material under compressive forces and elevating its axial stiffness^38^. Due to SF's higher rigidity and elasticity modulus and its end anchorage compared to PPF, SF has superior efficiency in improving all mechanical properties of such concretes. The presence of hooks at the ends of the SF gives it an exceptional ability to bond with the binder matrix compared to other fibers. In other words, SF keeps concrete from prematurely cracking under tension due to its ability to stop cracking^39^. Once again, these hooks help use the maximum fiber strength to resist tensile cracks by preventing fiber slippage. Failure due to slippage of fibers may occur in the case of plain fibers like PPF, even though their full strength in resisting tensile cracking has not been effectively utilized.

Many researchers have observed decreased concrete strength from using RU in the FRC mixes^23^. However, as Nguyan et al.^40^ established, SF relieves the harmful effects of the RU aggregates on tensile strength. However, when crumb RU was added, RFRC exhibited the same trend as regular concrete. The use of RU particles negatively impacted the flexural strength of concrete^9^. The bonding defect was noted in the RU aggregate size growth^41^. However, it was pointed out that the flexural strength of SF RFRC beams with a 1.28% SF volume fraction did not significantly improve over those with a 0.64% SF volume fraction^22^. In this regard, using SF can partially balance the problems of replacing sand with RU^42^. Adding SF and RU aggregate to concrete enhances its performance by slightly reducing its mechanical qualities while increasing its ability to absorb energy.

The strength ratio equation determines the enhancement of fiber additions to rubberized concrete as follows:

1 $$\mathrm{Strength \, ratio }= (1- \frac{\mathrm{strength \, of \, concrete \, with \, fibers }}{\mathrm{strength \, of \, concrete \,  without \, fibers}}) *100$$

The strength ratios of different FRC and RFRC mixes are listed in Table 2. In the case of SF, all strength ratios of different RFRCs increased with increasing the Vf%. The data in Table 2 reveals several points of interest:

- The strength ratio increased with increasing the Vf% of SF.
- The strength ratios of hybrid FRC and RFRC are higher than those of SF FRC and RFRC for the same Vf% of SF.
- It was observed that RFRC with Vf% = 1.5% SF has the highest compressive and tensile strength ratios for RU% = 25% and 5%, respectively. However, the highest flexural strength ratio was observed for hybrid FRC, i.e., RU% = 0.

**Table 2:** The effect of SF, PPF, and hybrid fibers on the compressive, tensile, and flexural strength ratios of RFRC.

RU%	Strength	SF0.5	SF1	SF1.5	PP0.4	SF1PP0.4
0%	Compressive	4.6	15.7	36.5	3.8	17.9
	Tensile	2.4	10.6	24.4	2.4	12.2
	Flexural	19.4	31.2	34.9	16.2	36.9
5%	Compressive	4.9	10.9	29.9	2.1	16.4
	Tensile	17.7	45.4	66.2	8.5	48.9
	Flexural	18.1	23.7	32.6	7.2	36.4
15%	Compressive	12	17.1	20.5	1.7	19.2
	Tensile	18.2	47.2	52	1.3	48.4
	Flexural	7.8	24.8	32.1	7	25.5
25%	Compressive	27.9	38.7	45.5	3	45
	Tensile	23.5	32.4	61.8	5.9	36.8
	Flexural	4.2	25.7	32.8	7.3	27.9

### Flexural behavior of RFRC

**Figure 11:**
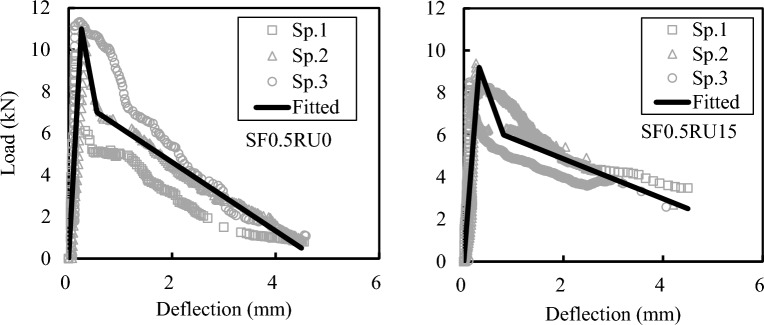
Experimental and fitted load–deflection curve for SF0.5RU0 and SF0.5RU15 beams.

Figure 11 represents samples of load–deflection curves for FRC and RFRC with 0.5Vf% of SF, i.e., SF0.5RU0 and SF0.5RU15. Each case has three tested specimens, as shown in the Figure. To fit the ascending and descending portions of the load–deflection curves for all beams, a multi-linear curve fitting technique^43^ was employed. The data from the three replicas for the SF0.5RU0 and SF0.5RU15 beams were fitted with a multi-linear solid curve, as demonstrated in Fig. 11. The fitted curve will be subsequently utilized to analyze the test results.

**Figure 12:**
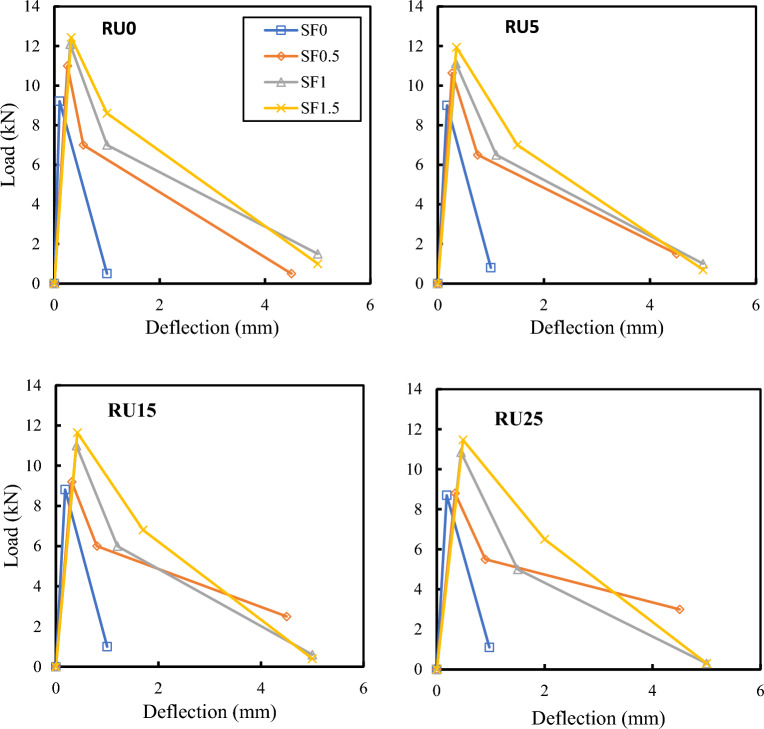
Effect of SF volume fraction on the load–deflection behavior at different RU%.

**Figure 13:**
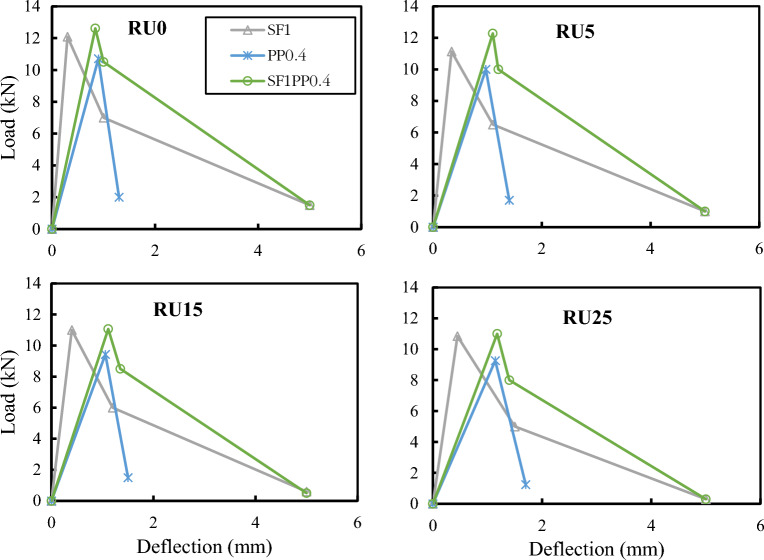
Effect of hybrid fiber on the load–deflection behavior at different RU%.

Figure 12 displays the influence of SF volume fraction on the load–deflection curve of RFRC at various RU ratios. Meanwhile, Fig. 13 illustrates the effect of PPF and hybrid fibers on the load–deflection curves of RFRC. Noticeably, including fibers leads to a marked improvement in the shape of the load–deflection curves, resulting in increased peak flexural load and corresponding deflection. In all cases where no fibers were present, the beams exhibited an almost linear behavior, with no observable cracks until the initiation of the first crack, which coincided with the maximum load. Depending on the presence of fibers, the load gradually decreases until it eventually fails. It can be observed that beams containing SF and PPF exhibit higher toughness. It can be inferred that fibers are more effective at bridging macro fractures than materials lacking fibers, thus displaying post-peak behavior. Consequently, it is deduced that adding fibers enhances the toughness of FRC beams, significantly impacting peak loads.

**Figure 14:**
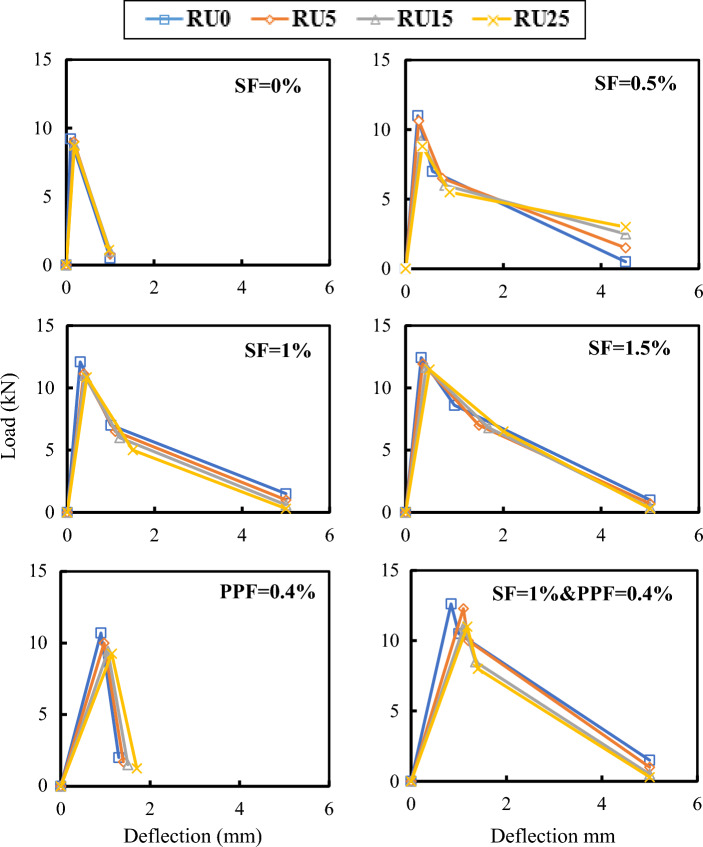
Effect of RU% on load–deflection of RFRC specimens.

The effect of RU replacement ratios on the load–deflection curves of RFRC at different volume fractions of fibers is depicted in Fig. 14. The curves were employed for estimating energy at peak load, fracture energy, and toughness indices, as presented in Table 3. The toughness indices were calculated per ASTM C1018^44^. An observable trend in the results is that the peak load decreases with an increase in RU percentage, whether in plain or RFRC, while deflection and energy at the peak load exhibit an increase. This phenomenon reflects the positive impact of RU and fibers in augmenting the energy capacity of the curves during the ascending loading phase.

**Table 3:** Toughness indices of concrete.

No.	Code	Deflection (mm)	Load (kN)	Energy at peak load (kN mm)	Fracture energy (kN mm)	Toughness indices		
						I5	I10	I20
1	Control (RU0)	0.16	9.21	0.75	4.83	4.3	6.2	7
2	RU5	0.17	9	0.79	4.83	4.3	6.4	6.4
3	RU15	0.18	8.81	0.8	4.82	4.3	6.3	6.3
4	RU25	0.19	8.7	0.81	4.68	4.2	5.8	6.2
5	SF0.5RU0	0.25	11	1.38	12.1	3.5	5.4	8.1
6	SF0.5RU5	0.27	10.63	1.44	11.9	3.7	5.3	7.6
7	SF0.5RU15	0.32	9.2	1.47	11.1	3.7	5.3	7.5
8	SF0.5RU25	0.34	8.8	1.5	10.6	3.5	4.9	7.3
9	SF1RU0	0.3	12.09	1.81	22.1	4.2	6.6	9.7
10	SF1RU5	0.35	11.13	1.95	21.4	4	6.4	9.7
11	SF1RU15	0.4	11	2.2	20.8	3.9	6	8.9
12	SF1RU25	0.45	10.85	2.44	18.8	3.7	5.5	7.6
13	SF1.5RU0	0.32	12.43	1.99	27.3	4.4	7.5	11.9
14	SF1.5RU5	0.35	11.94	2.1	25.6	4.5	7.6	11.3
15	SF1.5RU15	0.42	11.64	2.44	25.0	4.3	7.2	10.1
16	SF1.5RU25	0.49	11.47	2.8	24.6	4.1	6.6	9.2
17	PP0.4RU0	0.9	10.7	4.82	8.2	1.8	2.6	2.6
18	PP0.4RU5	0.97	10	4.85	7.9	2.1	2.1	2.1
19	PP0.4RU15	1.06	9.43	4.98	7.7	1.7	1.7	1.7
20	PP0.4RU25	1.14	9.27	5.29	7.4	1.4	1.4	1.4
21	SF1PP0.4RU0	0.84	12.62	5.3	31.1	4	5.1	5.1
22	SF1PP0.4RU5	1	12.28	6.14	28.8	3.7	4.6	4.6
23	SF1PP0.4RU15	1.11	11.3	6.29	24.9	3.8	4.8	4.8
24	SF1PP0.4RU25	1.18	11	6.49	21.1	3.4	4.2	4.2

Conversely, the data in Table 3 demonstrates that the toughness indices and fracture energy exhibit slight variations with increasing RU percentages but experience a marked increase with an increasing Vf%. Including PPF enhances the flexural energy at the peak load and fracture energy at all RU% compared to concrete mixes without fibers. The introduction of PPF alongside SF in the hybrid mix further enhances the energy capacity of the RFRC mix, both at the peak load and in terms of fracture energy. Increasing the SF Vf% also contributes to an expansion in the energy capacity of RUC beams. Toughness indices for RFRC were determined with various RU and fiber dosages. It was observed that the toughness indices of RFRC initially improved before declining as the dosage of fibers increased, reaching their maximum value of 0.75%. Additionally, it was found that RU and fibers impacted the flexural toughness of the concrete^45^. Steel fibers were found to significantly enhance the flexural toughness of the concrete, while RU particles had a minimal effect. Higher fiber dosages could lead to a dispersion issue, adversely affecting RFRC performance. Increased RU content in the concrete resulted in increased deformation (reduced stiffness) at the first crack but did not influence the toughness index.

The compressive, tensile, and flexural strengths of normal-strength concrete have been investigated in terms of their mutual effects with steel fiber and rubber. As discussed in numerous previous studies, it is essential to note that steel fiber has been introduced into normal-strength concrete to enhance its mechanical properties. The influence of steel fiber reinforcement on the compressive stress–strain behavior of normal-strength concrete produced from construction and demolition waste was examined by Carneiro et al.^46^. Their study revealed that the mechanical strength was increased, and the fracture process of the concrete was modified with the addition of steel fiber^46^. These observations are consistent with the present results. Furthermore, when steel fiber was added to rubberized concrete, the strength of such concrete was restored to that of concrete without rubber, i.e., resolving the negative effect of adding rubber on concrete strength, accompanied by additional energy absorption.

### Impact resistance of RFRC

The impact resistance of various types of concrete has been thoroughly examined in prior research studies, focusing on the distinct influence of either steel fibers or crumb rubber^47–53^. These investigations reported substantial enhancements, with improvements reaching several hundred percent. Specifically, the integration of hooked-end steel fibers demonstrated a significant increase in cracking resistance and a notable boost in impact resistance to failure. Despite these findings, limited research has explored the combined effect of steel fibers on the impact resistance of rubberized concrete. The present study examines the effect of adding steel fibers with different volume fractions with a rubber percentage of 15% to improve the impact resistance of such concrete, which will be discussed in this section.

All results of the number of blows that initiated the first visible crack (N_i_) and the number of blows at failure (N_f_) of both RUC and RFRC are listed in Table A2 and analyzed in Table 4. The evaluation of RFRC resistance was based on two parameters: the impact ductile index (μ_i_) and the impact energy (E). The extent of post-crack resistance and impact ductility was assessed by μ_i_, represented by the ratio of N_f_ to N_i_, denoted as N_f_/N_i_^47^. The calculation of impact energy was performed utilizing the following equation^35^:

2 $$ {\text{E }} = {\text{ N}} \cdot {\text{m}} \cdot {\text{v}}^{{2}} /{2} $$

**Table 4:** Impact test results.

Code	Impact ductility index (µ_i_)	Ratio to RUC	E_i_, kN mm	Ratio to RUC	E_f_, kN mm	Ratio to RUC
F0	1.002	1	24,910	1	24,971	1
SF0.5	1.008	1.006	80,736	3.24	81,374	3.26
SF1	1.011	1.009	131,056	5.26	132,560	5.31
SF1.5	1.015	1.013	165,262	6.63	167,783	6.72

The velocity of the hammer (v) can be calculated, and thus, the impact energy can be determined from the following Equation, where N is the number of blows, and the hammer mass (m) is 4.45 kg, with a falling height of 457 mm.

3 $$ {\text{E }} = { 2}0.{\text{33 N}}\;{\text{kN}}\;{\text{mm}} $$

**Figure 15:**
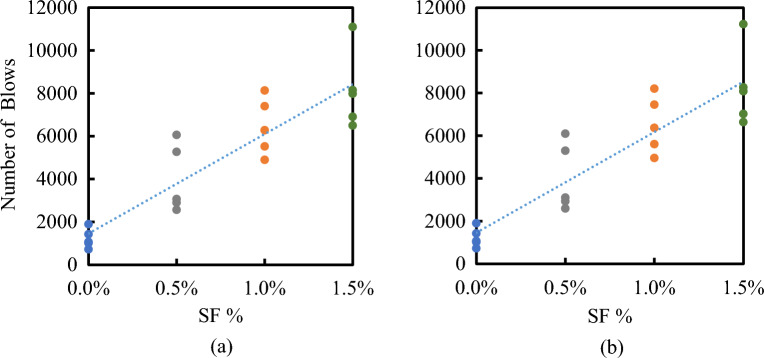
Effect of SF volume fraction of the number of blows at (**a**) First crack, (**b**) Final failure.

**Figure 16:**
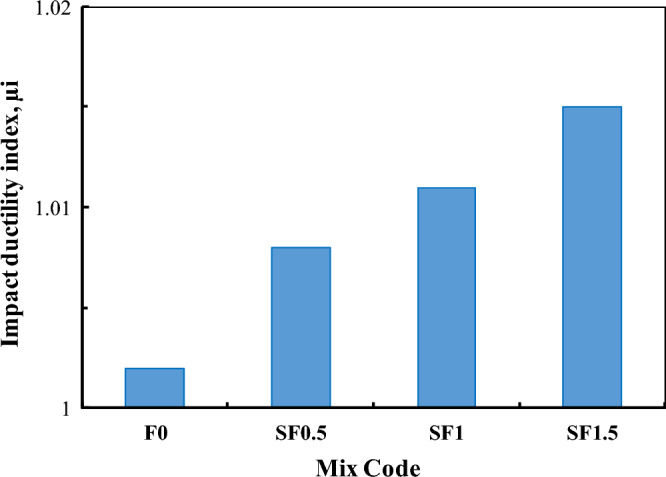
Impact ductile index µ_i_ of mixtures.

The impact resistance of SF RFRC of four selected mixes with RU% = 15% is shown in Fig. 15. It is clear that the scatter of the results of N_i_ and N_f_ of RUC, i.e., rubberized concrete without SF, is much lower than those of rubberized concretes with SF. This means that the randomization of rubber particles has a lower effect on the scattering of the impact results than the randomization of hocked-end steel fibers. In Table 4, the impact energy estimated at the first crack (E_i_), final failure (E_f_), and ductility index (µ_i_) is detailed. It is apparent that an increase in the Vf% of SF results in the impact energies and ductility index of RFRC. Notably, adding 0.5% to 1.5% SFs to rubberized concrete with 15% RU increased their impact energy from 3.25 to 6.7 times. The effect of adding SF on the impact ductility index is shown in Fig. 16. The Figure indicates an increase in µ_i_ with the incorporation of SF fibers in RUC. The presence of fibers enhanced the final impact resistance outcome due to their remarkable strength and stiffness. Consequently, fibers elevate the resistance to the first crack, as they can control cracks under impact loads. Despite the presence of initial cracks in the concrete specimen, it endured high-impact loads before succumbing to failure. These findings corroborate those reported in^35^.

### Fracture behavior under impact loads

**Figure 17:**
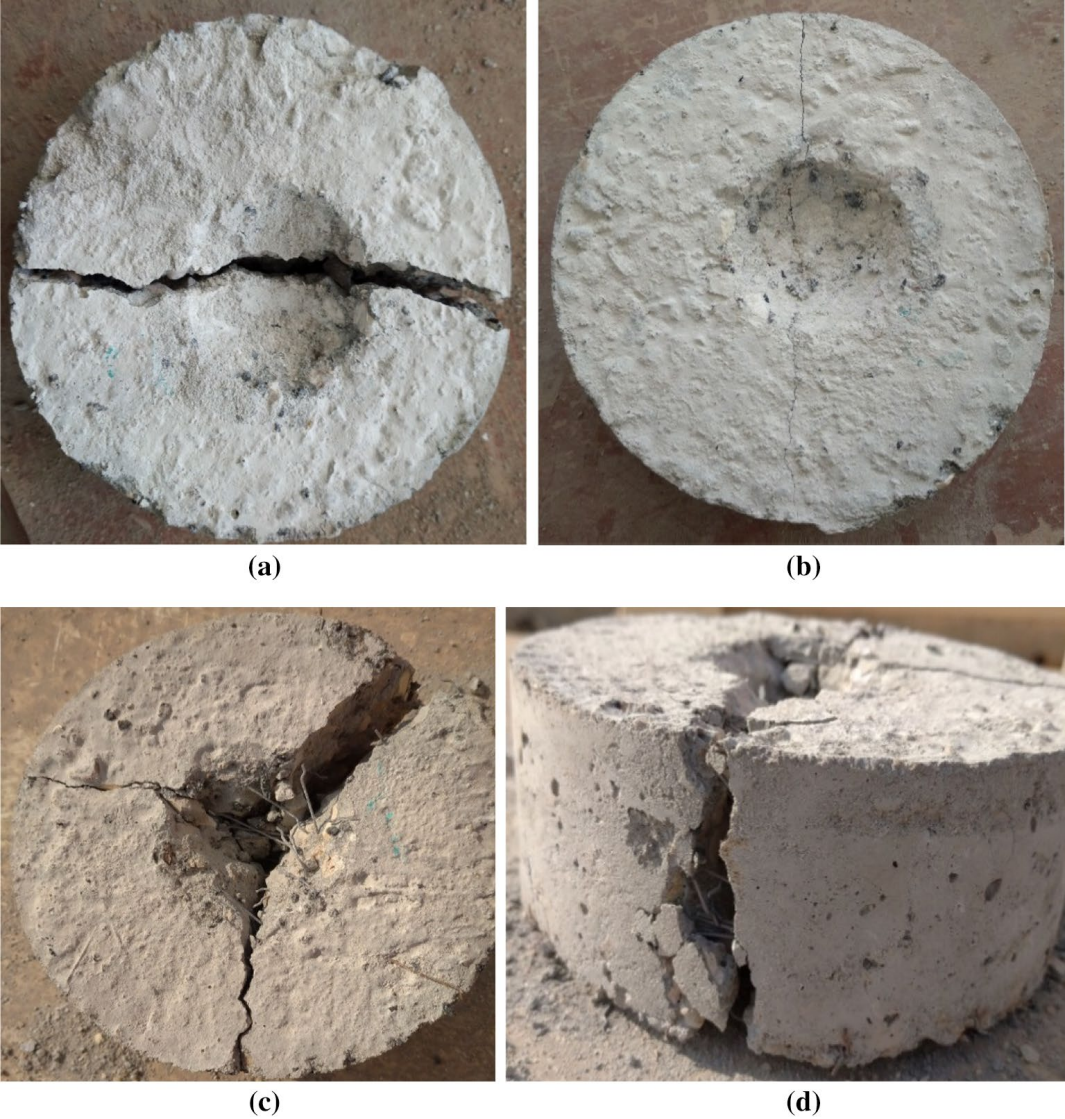
Failure patterns: (**a**) RUC failure, (**b**) RUC cracking, (**c**) RFRC main and secondary cracks, and (**d**) SF pullout.

The fracture characteristics of both RUC and RFRC specimens are shown in Fig. 17. It is noteworthy that RUC specimens exhibited notably lower impact strength compared to RFRC specimens. These RUC specimens tended to fail shortly after the initial appearance of the first fracture, indicating their brittleness when subjected to impact loads. Figure 17a visually represents a diagonal line crack formation in RUC as the cracking capacity was reached. This crack expanded after receiving one to three additional blows, eventually reaching the cylinder's edges, extending to the bottom surface, and causing the specimens to split in half. This brittle failure mode is the most frequently observed in RUC^36–38^. Additionally, it was observed that a minor fracture could occur before the ultimate failure, as depicted in Fig. 17b.

Due to the heterogeneous nature of concrete, cracks can form along paths that may be weaker than others, resulting in the initiation and growth of multiple cracks in various directions. In contrast, RFRC specimens, characterized by significantly improved impact resistance, exhibited distinct behavior. These specimens absorbed a higher number of impact blows, forming a central circular fracture beneath the impacting ball. The size of this zone increased as the number of impacts increased, causing surface splitting and the emergence of small surface cracks. Figure 17c displays the fracture surfaces of the fibrous sample. This crack extended to the outer perimeter and reached the bottom surface, progressively widening with increasing impact blows. As the bond between the fibers and the surrounding matrix rapidly weakened, the specimens failed due to fiber pullout, as shown in Fig. 17d. The fibers continued to connect both sides of the cracks. Several studies have shown that when reinforced with fibers, concrete transitions from brittleness to ductility when subjected to impact loading^39,40^. This transition occurs because of the bridging action of the fibers, which enhances the material's ductility, enabling it to absorb a greater amount of impact energy. Consequently, this process delays failure and increases the number of impacts the material can withstand after the initiation of cracking.

## Conclusions

The following conclusions are drawn from our experimental work, which investigates the impact of rubber content and fiber reinforcement on concrete's mechanical properties and energy absorption capabilities.

- RUC experienced a slight reduction in compressive strength at 5%RU, while a significant decrease was observed at higher RU%. On the other hand, the tensile strength substantially decreased for all RU%, while flexural strength slightly declined.
- In the case of RFRC, adding fibers led to significant enhancements in all mechanical properties, regardless of rubber content, especially steel fibers.
- The strength ratio increased with increasing the Vf% of SF. RFRC with Vf% = 1.5% SF has the highest compressive and tensile strength ratios for RU% = 25% and 5%, respectively. However, the highest flexural strength ratio was observed for hybrid FRC, i.e., RU% = 0. The strength ratios of hybrid FRC and RFRC are higher than those of SF FRC and RFRC for the same Vf% of SF.
- The toughness indices and fracture energy exhibit slight variations with increasing RU% but experience a marked increase with an increasing Vf%.
- Under static loads, the results highlight the potential of fiber reinforcement to counterbalance the adverse effects of rubber inclusion, improving the overall mechanical performance of rubberized concrete.
- In general, the impact energies and impact ductility index of RFRC increased with the increase in the Vf% of SF.
- Adding 0.5% to 1.5% SFs to rubberized concrete with 15% RU increased their impact energy from 3.25 to 6.7 times.

### Supplementary Information

Supplementary Tables.

## Data Availability

All data generated or analyzed during this study are included in this published article.
