# Supplementary material for: Effect of fiber addition on strength and toughness of rubberized concretes
Source: Sci Rep. 2024 Feb 22;14:4346. doi: 10.1038/s41598-024-54763-w (PMC10884028; doi:10.1038/s41598-024-54763-w)
Supplement: Supplementary file 1 — Supplementary Tables. [file 41598_2024_54763_MOESM1_ESM.docx]

**Appendix A**

**Table A1.** Compressive, tensile, and flexural strengths of all investigated mixes

| % age Rubber | | 0.0 | | 5% | | 15% | | 25% | |
| --- | --- | --- | --- | --- | --- | --- | --- | --- | --- |
| SF&PPF% | Strength | Strength (MPa) | C.O.V. | Strength (MPa) | C.O.V. | Strength (MPa) | C.O.V. | Strength (MPa) | C.O.V. |
| SF0 | Compressive | 26.0 | 0.06 | 25.3 | 0.05 | 21.5 | 0.033 | 16.5 | 0.08 |
|  | Tensile | 3.3 | 0.02 | 2.3 | 0.09 | 2.2 | 0.058 | 1.8 | 0.08 |
|  | Flexural | 3.7 | 0.06 | 3.6 | 0.04 | 3.5 | 0.028 | 3.5 | 0.02 |
| SF0.5 | Compressive | 27.2 | 0.03 | 26.6 | 0.04 | 24.1 | 0.076 | 21.1 | 0.04 |
|  | Tensile | 3.3 | 0.04 | 2.7 | 0.08 | 2.6 | 0.058 | 2.2 | 0.06 |
|  | Flexural | 4.4 | 0.02 | 4.3 | 0.06 | 3.8 | 0.054 | 3.6 | 0.06 |
| SF1 | Compressive | 30.1 | 0.08 | 28.1 | 0.09 | 25.2 | 0.041 | 22.9 | 0.05 |
|  | Tensile | 3.6 | 0.04 | 3.3 | 0.07 | 3.2 | 0.061 | 2.4 | 0.03 |
|  | Flexural | 4.8 | 0.07 | 4.5 | 0.07 | 4.4 | 0.038 | 4.3 | 0.03 |
| SF1.5 | Compressive | 35.5 | 0.04 | 32.9 | 0.10 | 25.9 | 0.058 | 24.0 | 0.07 |
|  | Tensile | 4.1 | 0.03 | 3.8 | 0.06 | 3.3 | 0.099 | 2.9 | 0.09 |
|  | Flexural | 5.0 | 0.08 | 4.8 | 0.03 | 4.7 | 0.034 | 4.6 | 0.03 |
| PP0.4 | Compressive | 27.0 | 0.07 | 25.0 | 0.08 | 21.9 | 0.071 | 17.0 | 0.05 |
|  | Tensile | 3.3 | 0.07 | 2.5 | 0.09 | 2.2 | 0.058 | 1.9 | 0.10 |
|  | Flexural | 4.3 | 0.08 | 3.9 | 0.07 | 3.8 | 0.083 | 3.7 | 0.05 |
| SF1PP0.4 | Compressive | 30.7 | 0.03 | 29.5 | 0.06 | 26.4 | 0.090 | 24.0 | 0.07 |
|  | Tensile | 3.7 | 0.04 | 3.4 | 0.02 | 3.3 | 0.024 | 2.5 | 0.03 |
|  | Flexural | 5.0 | 0.08 | 4.9 | 0.09 | 4.6 | 0.118 | 4.4 | 0.17 |

**Table A2.** Number of blows at first crack and failure of RUC and RFRC

| Mix Code | Spec. No. | N_i_ blows | Mean at first crack | S.D | C.O.V. | N_f_ blows | Mean At failure | S.D | C.O.V. |
| --- | --- | --- | --- | --- | --- | --- | --- | --- | --- |
| RU 15% | 1 | 725 | 1225 | 450 | 3 | 728 | 1228 | 450 | 3 |
|  | 2 | 1066 |  |  |  | 1069 |  |  |  |
|  | 3 | 1425 |  |  |  | 1426 |  |  |  |
|  | 4 | 1015 |  |  |  | 1018 |  |  |  |
|  | 5 | 1895 |  |  |  | 1900 |  |  |  |
| SF0.5RU15 | 1 | 2565 | 3971 | 1579 | 3 | 2590 | 4002 | 1583 | 3 |
|  | 2 | 6055 |  |  |  | 6094 |  |  |  |
|  | 3 | 5270 |  |  |  | 5300 |  |  |  |
|  | 4 | 2900 |  |  |  | 2928 |  |  |  |
|  | 5 | 3065 |  |  |  | 3100 |  |  |  |
| SF1RU15 | 1 | 8131 | 6446 | 1326 | 5 | 8206 | 6520 | 1325 | 5 |
|  | 2 | 6278 |  |  |  | 6372 |  |  |  |
|  | 3 | 7401 |  |  |  | 7458 |  |  |  |
|  | 4 | 4897 |  |  |  | 4957 |  |  |  |
|  | 5 | 5523 |  |  |  | 5607 |  |  |  |
| SF1.5RU15 | 1 | 11100 | 8128 | 1802 | 5 | 11233 | 8252 | 1803 | 5 |
|  | 2 | 6502 |  |  |  | 6642 |  |  |  |
|  | 3 | 6905 |  |  |  | 7027 |  |  |  |
|  | 4 | 8150 |  |  |  | 8270 |  |  |  |
|  | 5 | 7985 |  |  |  | 8090 |  |  |  |
| SF1PP0.4RU15 | 1 | 3300 | 4592 | 1135 | 4 | 3370 | 4680 | 1162 | 4 |
|  | 2 | 4250 |  |  |  | 4327 |  |  |  |
|  | 3 | 3920 |  |  |  | 3980 |  |  |  |
|  | 4 | 6100 |  |  |  | 6236 |  |  |  |
|  | 5 | 5390 |  |  |  | 5487 |  |  |  |
